# Comparative cardiac electrophysiological analysis between sinus rhythm and atrial fibrillation: The correlation of left atrial low-voltage substrate of sex and rhythm

**DOI:** 10.1016/j.hroo.2025.11.026

**Published:** 2025-12-05

**Authors:** Guifang Zhao, Xuefeng Zhu, Pingping Liu, Faxin Ren, Ronghang Liu, Yanyan Jing, Hongxia Chu

**Affiliations:** 1 School of Clinical Medicine, Qingdao University, Qingdao, Shandong, P.R. China; 2 Department of Cardiology, The Affiliated Yantai Yuhuangding Hospital of Qingdao University, Yantai, Shandong, P.R. China; 3 Department of Statistics, Binzhou Medical University, Yantai, P.R. China

**Keywords:** Atrial fibrillation, Low-voltage areas, Electroanatomic mapping, Sex, Sinus rhythm

## Abstract

**Background:**

It is uncertain whether bipolar voltage cutoffs should be adjusted based on rhythm, sex, or region.

**Objective:**

This study aimed to determine (1) the bipolar voltage cutoff values for the entire left atrium (LA) and the low-voltage areas in different regions of the LA and (2) whether sex-based differences exist in the bipolar voltage cutoff points for low-voltage areas.

**Methods:**

30 patients with persistent atrial fibrillation (AF) underwent high-density voltage mapping first in sinus rhythm and subsequently during induced AF. The 7 parts of the map are respectively taken from 9 regions with the same position and location. The average low voltage of these area points was analyzed. Correlation was assessed using scatter plots, and agreement was evaluated with Bland-Altman analysis. The generalized additive model was used to predict the bipolar voltage cutoff points.

**Results:**

A total of 1268 regions were obtained. For the entire LA, a cutoff of 0.27 mV in AF predicted a sinus rhythm voltage of 0.5 mV (95% confidence interval 0.12–2.02). Region-specific AF voltage cutoffs were as follows: posterior wall 0.19 mV, inferior wall 0.17 mV, anterior wall 0.27 mV, LA appendage 0.27 mV, and roof 0.45 mV. Regarding different sexes, the values were 0.13 mV for males and 0.32 mV for females, respectively.

**Conclusion:**

It is possible to establish a new cutoff value for AF with acceptable validity in predicting sinus voltage of <0.5 mV, but sex differences need to be taken into account.

**Key Findings**

▪ In a sex-balanced cohort, we established sinus rhythm (SR) low-voltage area cutoffs from atrial fibrillation (AF) maps and quantified sex-specific voltage differences.
▪ The following 3 findings emerged: (1) global mean left atrial bipolar voltage was markedly higher in SR than AF, (2) women had lower voltages than men at most sites in either rhythm, and (3) AF thresholds (0.27 mV overall; 0.13 mV in men, 0.32 mV in women) predicted an SR of <0.5 mV with acceptable accuracy.

## Introduction

Success rates of pulmonary vein isolation alone for persistent atrial fibrillation (AF) are significantly lower than those for paroxysmal AF, possibly because additional arrhythmogenic atrial sites are responsible for AF maintenance. Atrial fibrosis and scar tissue constitute the pathologic substrate that critically perpetuates persistent.^1^, ^2^, ^3^ Therefore, low-voltage areas (LVAs) substrate modification has been performed to improve long-term AF ablation efficacy, and most of these procedures have performed mapping using established cutoffs for voltage during sinus rhythm (SR), except for 1 study, which performed mapping during AF.^4^, ^5^, ^6^, ^7^

There remain multiple unresolved issues with LVA-guided substrate modification during SR. First, at the same voltage cutoff, the LVAs during SR are different from those mapped during AF.^8^^,^^9^ Second, mapping in SR is not always feasible; because of repeated/persistent AF-triggering activity, AF reoccurs shortly after cardioversion. Moreover, recent studies have demonstrated that LVAs identified during AF may serve as a more accurate indicator of potentially persistent AF substrates than low-voltage zones detected during SR.^8^^,^^10^^,^^11^ Some electrophysiologists may choose to perform mapping during AF to identify both the potential arrhythmogenic rapid trigger sites and the underlying pathologic substrate.^10^^,^^12^ Therefore, it is important to determine the voltage correlation between SR and AF. Some studies have established new cutoffs for AF with sinus voltage of <0.5 mV, but the corresponding cutoffs for AF are inconsistent.^9^^,^^13^ The possible reasons are the differences in statistical methods and the proportion of male and female participants, given that some studies have found sex-based differences in electrophysiological substrates.^14^ Furthermore, whether bipolar voltage cutoffs should vary with rhythm, sex, or mapped region remains unclear.

We aimed to (1) determine the voltage correlation between SR and AF, (2) determine the differences in voltage between men and women under different rhythms and regions, and (3) identify the bipolar voltage cutoff for LVAs during AF for different genders and different regions, respectively, corresponding to an SR bipolar voltage of 0.5 mV.

## Methods

This study was a single-center, retrospective investigation. From January 2023 to January 2024, patients who underwent an initial ablation for persistent AF at Yantai Yuhuangding Hospital were consecutively enrolled. Exclusion criteria were as follows: age <18 years, previous cardiac surgery, previous catheter ablation, pacemaker implantation, or severe mitral valve disease. In addition, patients in whom induced AF was not sustained during voltage mapping were excluded. To evaluate whether there are sex-based differences in bipolar voltage cutoff points within LVAs, an additional 1:1 propensity score matching between males and females was performed. 30 patients with persistent AF presenting for their first AF ablation procedure were included in the study. This study was conducted in accordance with the Declaration of Helsinki. A written informed consent for the ablation and participation in the study was obtained from all patients, and the protocol was approved by our institutional review board.

### High-density electroanatomic mapping

In all patients, antiarrhythmic medications were discontinued for at least 5 half-lives before the procedure. Transesophageal echocardiography was performed within 48 hours before the procedure to exclude left atrial (LA) thrombus. All procedures were performed under general anesthesia. High-density voltage mapping was performed using the CARTO 3 mapping system and PentaRay catheter (2-mm interelectrode spacing, Biosense Webster). The detailed high-density electroanatomic mapping protocol is presented in Supplemental Material 1.

**Figure 1:**
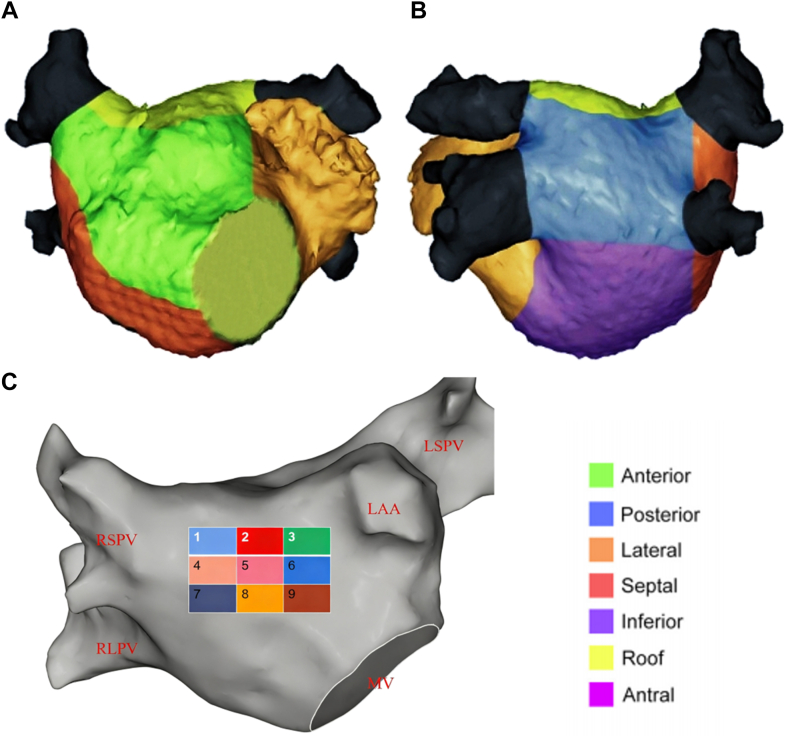
Regional analysis of the atrium. The atrial surface was subdivided into smaller, identical sections to facilitate the quantification of low-voltage areas. Anteroposterior (**A**) and posteroanterior (**B**) views of the left atrium divided into 7 segments: anterior, posterior, inferior, lateral, septal, roof, and inferior walls for regional analysis. The anterior region was divided into 9 segments on each side for subsegmental analysis of voltage (**C**). LAA = left atrial appendage; LSPV = left superior pulmonary vein; MV = mitral valve; RLPV = right lower pulmonary vein; RSPV = right superior pulmonary vein.

To compare SR and AF voltage maps and identify optimal regional thresholds, areal voltage analysis was performed. The LA was divided into 7 regions: anterior, inferior, septal, lateral, appendage, posterior, and roof (Figure 1); pulmonary vein and mitral annulus points were excluded. For each patient, 2 bipolar voltage maps (SR and AF) were created. From every region, 9 6 × 4 mm rectangles were sampled, and voltages were recorded in both rhythms. Finally, patients were separated by sex, thresholds were derived independently, and gender-specific differences were analyzed.

### Statistical analysis

Numerical data were tested for normality using the Shapiro-Wilk test and for homoskedasticity with Levene’s test, then summarized with mean, standard deviations, and interquartile range values where appropriate (interquartile range). Correlation and agreement were assessed by scatter and Bland-Altman (Tukey mean difference) plots. The generalized additive model (GAM) predicted the SR voltage, equivalent to a new set of simulated AF voltages; 0.5 mV peak to peak in SR served as the scar threshold. The AF thresholds were defined as the peak-to-peak voltage equivalent to a sinus amplitude of 0.5 mV and were set to be the corresponding values when the predicted sinus value matched 0.5 mV. To evaluate model performance, we applied a GAM to predict SR bipolar voltage from the corresponding AF voltage at each mapped point. Predicted SR voltages were then dichotomized (<0.5 mV or ≥0.5 mV) and compared with the observed SR voltages at the same sites. This comparison allowed calculation of positive predictive value (PPV), negative predictive value (NPV), sensitivity, specificity, and overall accuracy. All analyses were performed using R software, using the packages readxl, ggplot2, dplyr, ggridges, scales, BlandAltmanLeh, and mgcv.

## Results

### Patient characteristics

**Figure 2:**
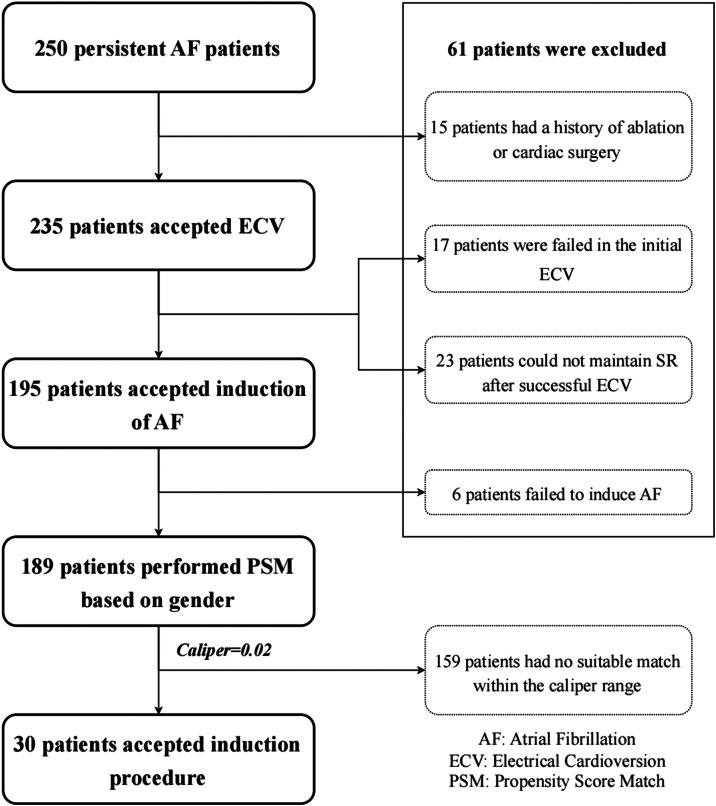
Flowchart of this study. AF = atrial fibrillation; ECV = electrical cardioversion; PSM = propensity score match; SR = sinus rhythm.

The study flow is presented in Figure 2. Between January 2023 and January 2024, 250 patients with persistent AF scheduled for catheter ablation at Yantai Yuhuangding Hospital were screened; 61 were excluded (10 previous AF ablation, 5 cardiac surgery, 17 failed cardioversion, 23 no SR maintenance, 6 noninducible AF), leaving 134 men and 55 women who fulfilled all criteria. Baseline data are presented in Supplemental Table 1.

After propensity score matching, the study included 30 patients (67.5 [62–70.3]) with persistent AF who were initially undergoing ablation procedures for AF. Baseline characteristics of the patients are presented in Table 1.

**Table 1:** Baseline characteristics after PS matching

Parameters	Total (n = 30)	Females (n = 15)	Males (n = 15)	*P* value
Age, y	67.5 (62–70.3)	68 (62–71)	67 (62–69)	.739
BMI, kg/m^2^	27.76 (25.17–28.98)	27.89 (24.54–29.39)	27.38 (25.31–28.73)	.787
Hypertension, n (%)	16 (53.3)	8 (53.3)	8 (53.3)	1
Diabetes, n (%)	7 (23.3)	4 (26.7)	3 (20.0)	1
Stroke, n (%)	3 (10.0)	2 (13.3)	1 (6.7)	1
Structural heart disease, n (%)	0	0	0	1
CHD, n (%)	7 (23.3)	4 (26.7)	3 (20.0)	1
AF duration, mo	2.5 (1–19.75)	3 (1–12)	2 (1–48)	.861
CHA_2_DS_2_-VASc score	3 (1.75–3.25)	3 (2–3)	2 (1–4)	.654
HAS-BLED score	1 (1–2)	2 (1–2)	1 (1–2)	.758
LAD, mm	44.64 ± 5.22	44.54 ± 6.73	44.74 ± 3.35	.919
LVED, mm	46.12 ± 5.49	46.35 ± 5.73	45.89 ± 2.97	.782
LVEF, %	62.5 (56.75–66)	62 (56–66)	63 (57–66)	.835
BNP, pg/mL	178.02 (99.21–314.03)	180.31 (114.69–335.98)	174.56 (87.57–238.01)	.494
CrCL, mL/min/1.73 m^2^	93.77 (87.29–105.53)	89.6 (87.29–104.82)	97.74 (91.4–116.95)	.206

AF = atrial fibrillation; BMI = body mass index; BNP = brain natriuretic peptide; CHD = coronary heart disease; CrCL = creatinine clearance; LAD = left atrial diameter; LVED = left ventricular end-diastolic diameter; LVEF = left ventricular ejection fraction.

All patients underwent sequential maps in SR and AF. There were significantly more points collected in the AF group (2536 ± 417 points vs 2257 ± 371 points; *P* < .01). There is no significant difference in each subregion. In total, 1268 pairs of electrograms during SR and AF were obtained. The number of point counts and anatomic distributions is presented in Supplemental Table 2.

### Comparison of voltages

The mean regional LA voltage distribution of SR and AF is presented in Table 2 and Supplemental Figure 1. Mean global voltage was higher during SR than that during AF (1.058 ± 1.013 vs 0.678 ± 0.684 mV; *P* < .001). From a regional perspective, this difference also exists, with significantly lower mean voltages in the anterior (0.992 ± 0.761 mV vs 0.613 ± 0.545 mV; *P* < .001), posterior (1.095 ± 0.923 mV vs 0.478 ± 0.437 mV; *P* < .001), lateral (1.359 ± 1.211 mV vs 0.707 ± 0.606 mV; *P* < .001), inferior (1.299 ± 1.339 mV vs 0.601 ± 0.504 mV; *P* < .001), and roof segments (0.888 ± 0.984 mV vs 0.648 ± 0.662 mV; *P* < .001).

**Table 2:** Mean regional and gender left atrial voltage distribution in sinus rhythm and atrial fibrillation rhythm

Region	Sinus rhythm	AF rhythm	*P* value
Overall	1.058 ± 1.013	0.678 ± 0.684	<.001
Anterior	0.922 ± 0.761	0.613 ± 0.545	<.001
Posterior	1.095 ± 0.923	0.478 ± 0.437	<.001
Lateral	1.359 ± 1.211	0.707 ± 0.606	<.001
Septum	0.699 ± 0.630	0.599 ± 0.633	.072
Inferior	1.299 ± 1.339	0.601 ± 0.504	<.001
Roof	0.888 ± 0.984	0.648 ± 0.662	<.001
Left atrial appendage	1.409 ± 1.124	1.328 ± 1.107	.512
Male	1.250 ± 1.126	0.779 ± 0.655	<.001
Anterior	1.109 ± 0.076	0.799 ± 0.06	<.001
Posterior	1.271 ± 0.092	0.521 ± 0.033	<.001
Lateral	1.585 ± 0.152	0.819 ± 0.068	<.001
Septum	0.732 ± 0.073	0.72 ± 0.073	.890
Inferior	1.528 ± 0.164	0.671 ± 0.054	<.001
Roof	1.07 ± 0.112	0.706 ± 0.046	.001
Left atrial appendage	1.693 ± 0.14	1.335 ± 0.107	.024
Female	0.847 ± 0.822	0.566 ± 0.699	<.001
Anterior	0.723 ± 0.056	0.414 ± 0.025	<.001
Posterior	0.892 ± 0.078	0.429 ± 0.051	<.001
Lateral	1.072 ± 0.111	0.565 ± 0.07	<.001
Septum	0.666 ± 0.061	0.474 ± 0.059	.008
Inferior	1.106 ± 0.153	0.542 ± 0.063	.001
Roof	0.700 ± 0.067	0.589 ± 0.078	.183
Left atrial appendage	0.973 ± 0.096	1.317 ± 0.182	.097

In contrast, the septum remained relatively unchanged. In both rhythms, the highest voltages were observed in the LA appendage (LAA), and the voltage changes between the 2 were not significant.

Comparable results were observed in both genders: bipolar atrial voltage was significantly higher during SR than during AF in men (1.250 ± 1.126 mV vs 0.779 ± 0.665 mV; *P* < .01) and in women (0.847 ± 0.822 mV vs 0.566 ± 0.699 mV; *P* < .01). In male patients, significant voltage differences between SR and AF were observed except in the septum and LAA, whereas, in female patients, such differences were found except in the septum, LAA, and roof (Table 2).

During SR, bipolar atrial voltage differed significantly between the following pairs: anterior vs posterior, anterior vs lateral, anterior vs septum, anterior vs inferior, anterior vs LAA, posterior vs lateral, posterior vs septum, posterior vs roof, posterior vs LAA, lateral vs septum, lateral vs roof, septum vs inferior, septum vs roof, septum vs LAA, inferior vs roof, and roof vs LAA (all *P* < .05). During AF, significant differences were likewise present between anterior and posterior, anterior and LAA, posterior and lateral, posterior and septum, posterior and inferior, posterior and roof, posterior and LAA, lateral and LAA, septum and LAA, inferior and LAA, and roof and LAA (all *P* < .05). Regional differences for each rhythm are presented in Table 3.

**Table 3:** Regional differences obtained in sinus rhythm and AF

Region pairs	Sinus rhythm	AF rhythm
Anterior and posterior	0.029	0.004
Anterior and lateral	<0.001	0.116
Anterior and septum	0.002	0.803
Anterior and inferior	0.001	0.831
Anterior and roof	0.681	0.534
Anterior and LAA	<0.001	<0.001
Posterior and lateral	0.026	<0.001
Posterior and septum	<0.001	0.033
Posterior and inferior	0.088	0.015
Posterior and roof	0.025	0.002
Posterior and LAA	0.007	<0.001
Lateral and septum	<0.001	0.119
Lateral and inferior	0.692	0.108
Lateral and roof	<0.001	0.392
Lateral and LAA	0.719	<0.001
Septum and inferior	<0.001	0.970
Septum and roof	0.022	0.450
Septum and LAA	<0.001	<0.001
Inferior and roof	0.001	0.445
Inferior and LAA	0.465	<0.001
Roof and LAA	<0.001	<0.001

AF = atrial fibrillation; LAA = left atrial appendage.

### Comparison between males and females

**Figure 3:**
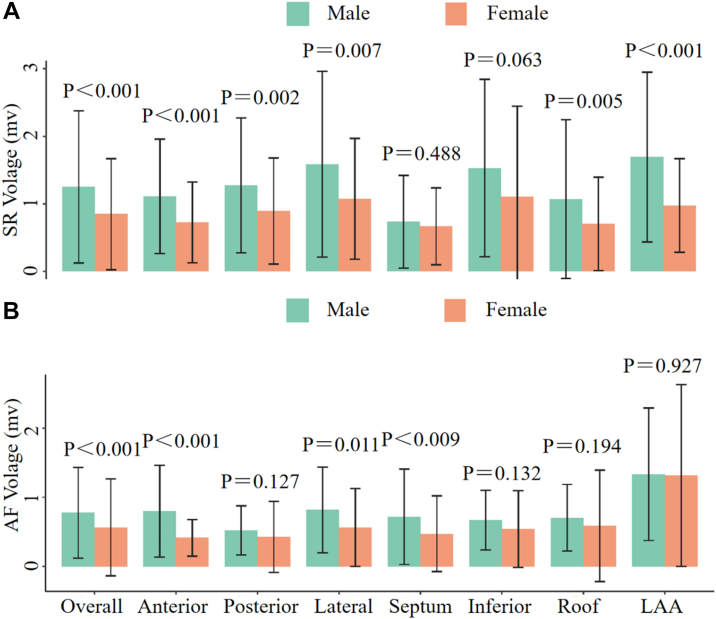
The difference between men and women with the same heart rhythm and location. **A:** The overall left atrial voltage in men with SR was higher than that in women, and there was no significant difference in the septum and inferior parts. **B:** The overall left atrial voltage in men with atrial fibrillation was also higher than that in women, and there was no significant difference in the posterior inferior roof and LAA parts. AF = atrial fibrillation; LAA = left atrial appendage; SR = sinus rhythm.

Baseline characteristics were comparable between sexes in each group. The significant disparity between men and women within the same heart rhythm is presented in Figure 3. Whether SR or AF, the voltage of most parts in women is lower than that in men. Compared with females, males exhibited significant differences in the anterior, posterior, lateral, roof, and LAA regions—except for the septum (*P* = .448) and inferior wall (*P* = .063)—under SR, whereas, under AF rhythm, differences were confined to the anterior (*P* < .001), lateral (*P* = .011), and septum regions (*P* < .009).

### Voltage correlation between SR–AF and different genders with a potential cutoff value for LVAs

**Figure 4:**
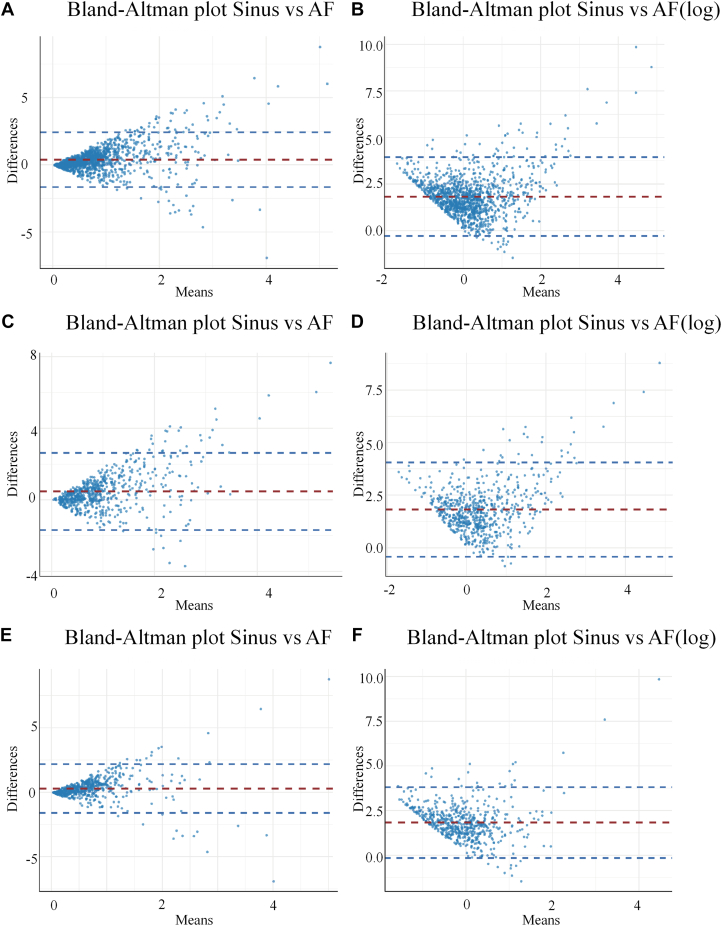
Bland-Altman plots showing the agreement between voltages in SR and AF **(A)** among all patients, as well as voltages across genders, male **(C)** or female **(E)**. Natural logarithmic scales **(B, D, and F)** are shown. AF = atrial fibrillation; SR = sinus rhythm.

After Bland-Altman analysis, the corresponding plots in Figure 4A, 4C, and 4E revealed a pronounced heteroscedastic funnel-shaped pattern. After natural logarithmic transformation, this heteroscedasticity was corrected, as shown in Figure 4B, 4D, and 4F, with 95% of the data points lying within the limits of agreement, indicating good concordance.

**Figure 5:**
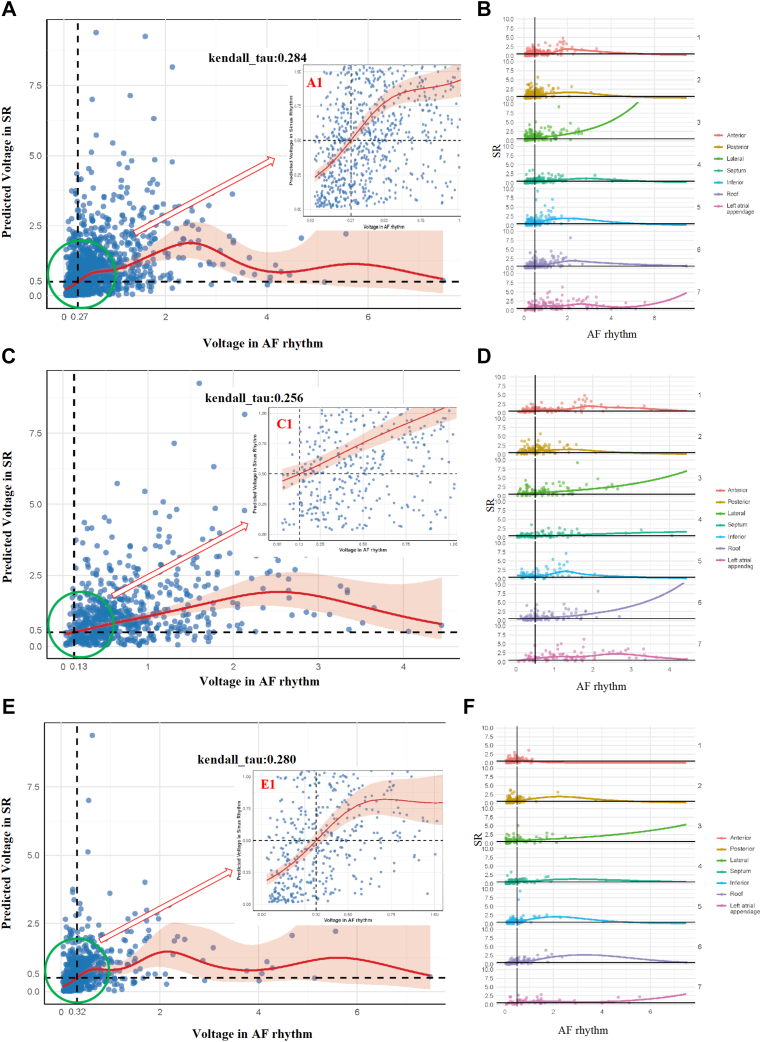
Linear and graphical bipolar voltage correlation between the different rhythms. **A:** Linear bipolar voltage correlation between AF and SR, A1 display cutoff value is 0.27 mV. **B:** Comparison of regional bipolar voltage correlation. **C:** Linear bipolar voltage correlation between AF and SR of man, C1 display cutoff value is 0.13 mV. **D:** Comparison of regional bipolar voltage correlation. **E:** Linear bipolar voltage correlation between AF and SR of woman, E1 display cutoff value is 0.32 mV. **F:** Comparison of regional bipolar voltage correlation. AF = atrial fibrillation; SR = sinus rhythm.

Scatter plots and Kendall’s tau coefficients were used to quantify the association between the 2 variables. As illustrated in Figure 5A, 5C, and 5E, Kendall’s tau coefficients were moderate, indicating a discernible correlation between AF voltage and SR voltage. Notably, when AF voltage was <0.5 mV, the correlation between AF and SR voltages seemed stronger; however, this approach excludes higher-voltage data points. A graphical comparison of regional bipolar voltage correlations is presented in Figure 5B, 5D, and 5F.

In Figure 5 and Table 4, GAMs were used to derive, for each discrete LA point acquired during AF, the predicted SR bipolar voltage together with its 95% confidence interval (CI). For the overall cohort, an AF voltage of 0.27 mV corresponded to an SR voltage of 0.5 mV (95% CI 0.456–0.533), with an NPV of 0.765, PPV of 0.536, specificity of 0.824, sensitivity of 0.446, and overall accuracy of 0.706. Among man, an AF voltage of 0.13 mV predicted an SR voltage of 0.5 mV (95% CI 0.430–0.582) with a specificity of 0.938, sensitivity of 0.420, and overall accuracy of 0.812, whereas in women an AF voltage of 0.32 mV predicted the same SR threshold (95% CI 0.450–0.568) with a specificity of 0.709, sensitivity of 0.613, and overall accuracy of 0.672. Details on the PPV, NPV, specificity, sensitivity, and accuracy for the entire population and for each predefined LA region, stratified by sex, are presented in Table 4. As also shown in Supplemental Table 3, 0.27 mV in atrial fibrillation would correspond with 0.5 mV in sinus rhythm (95% CI 0.078–1.578 and 0.052–1.198, respectively).

**Table 4:** Global and regional final generalized additive models

Region	Volage (mV)	NPV	PPV	Specificity	Sensitivity	Accuracy
Overall						
Sinus-AF	0.27	0.765	0.536	0.824	0.446	0.706
Anterior	0.27	0.798	0.569	0.831	0.514	0.735
Posterior	0.19	0.784	0.521	0.854	0.403	0.726
Lateral		0.776	NA	1.000	0.000	0.776
Septum	0.57	0.742	0.564	0.505	0.785	0.631
Inferior	0.06	0.793	NA	1.000	0.000	0.793
Roof	0.45	0.786	0.697	0.754	0.734	0.745
Left atrial appendage	0.27	0.818	0.545	0.952	0.714	0.795
Men						
Sinus-AF	0.13	0.769	0.609	0.982	0.686	0.764
Anterior	0.19	0.843	0.500	0.960	0.582	0.821
Posterior	0.06	0.807	1.000	1.000	0.620	0.812
Lateral		0.829	NA	1.000	0.000	0.829
Septum	0.83	0.710	0.569	0.468	0.786	0.618
Inferior		0.875	NA	1.000	0.000	0.875
Roof	0.38	0.720	0.607	0.843	0.425	0.691
Left atrial appendage	0.15	0.873	1.000	1.000	0.891	0.875
Women						
Sinus AF	0.32	0.741	0.574	0.709	0.613	0.672
Anterior	0.32	0.688	0.588	0.677	0.600	0.643
Posterior	0.29	0.727	0.468	0.615	0.595	0.608
Lateral		0.708	NA	1.000	0.000	0.708
Septum	0.4	0.794	0.566	0.540	0.811	0.655
Inferior	0.23	0.774	0.500	0.873	0.333	0.724
Roof	0.48	0.837	0.746	0.692	0.870	0.783
Left atrial appendage	0.35	0.738	0.600	0.886	0.653	0.712

AF = atrial fibrillation; NA = not available; NPV = negative predictive value; PPV = positive predictive value.

S.

## Discussion

This is the first study to establish SR–AF voltage cutoffs in a 1:1 sex-matched cohort and to examine sex differences in atrial voltage in persistent AF. Our main findings were as follows: (1) there were significant differences in global and regional voltage distribution when we compared different areas during the same rhythm or different rhythms in the same area; (2) despite a difference in voltage between rhythms, it was possible to establish new cutoffs for AF with acceptable validity in predicting a sinus voltage <0.5 mV; and (3) atrial voltage was significantly lower in women than in men, leading to sex-specific bipolar voltage cutoffs. These observations have clinical implications for LA voltage mapping, especially when scar is used to guide catheter ablation for AF.

### Relationship between atrial fibrosis and AF

Although current data demonstrate a robust association between atrial fibrosis and AF, the precise mechanisms by which fibrosis initiates or perpetuates AF remain incompletely understood. Atrial fibrosis has been observed in greater frequency in patients with persistent AF.^1^^,^^15^^,^^16^ At the same time, evidence for extensive fibrosis is linked to longer arrhythmia history and lower pulmonary vein isolation success rates. This implies a possible mechanistic link between atrial fibrosis and/or scarring and AF. The DECAAF study demonstrated a significant correlation between atrial fibrosis extent and AF recurrence risk. The quantitative assessment of atrial fibrosis in patients showed that, for every 1% increase in fibrosis degree, the risk of AF recurrence increased by 6%. Manipulating the substrate in fibrotic areas may enhance outcomes for some patients.^6^^,^^17^ However, the potential benefits of ablation for LVAs remain unclear, and recent studies have yielded different results.^4^, ^5^, ^6^^,^^18^^,^^19^

Although fibrosis is a recognized indicator of arrhythmogenic substrate, not all fibrotic areas necessarily harbor reentrant circuits or contribute directly to AF perpetuation. Hence, the difficulty in distinguishing between fibrotic areas that actively sustain arrhythmias and those that do not remains a conflicting issue. Previous studies suggest that LVAs observed during AF may serve as a more accurate indicator of potentially persistent AF substrates than low voltages detected during SR.^8^^,^^20^

Studies have shown that rotors involved in AF maintenance exhibited lower voltage than sites without rotors.^21^^,^^22^ During SR, the underlying AF substrate is in an electrophysiologically passive state, featuring low rates and coordinated activation wavefronts. Under these conditions, low voltage is known to result as the propagating wavefront encounters conduction barriers associated with compact fibrosis. In this passive state, noncompact fibrotic regions susceptible to arrhythmogenic activity may lie dormant. During AF, activation rates are more rapid, and nontransmural or patchy fibrotic tissue may be more susceptible to functional reentry, slow conduction, or conduction block, which results in LVAs. Whether performing LVAs ablation during AF rhythm improves long-term procedural success remains controversial and will require further mechanistic and randomized investigation.

### Difference in SR and AF of cutoff value

Our finding of higher voltages in SR than AF is in agreement with previous studies in the literature. Yagishita et al^23^ found a linear voltage correlation between SR and AF, with lower voltages in AF than in SR. During arrhythmias with shorter cycle lengths, a substantial amount of tissues might not depolarize when it is still refractory, or small pieces of underlying or neighboring tissues might depolarize nonsimultaneously and in opposite directions, which results in low bipolar voltage amplitudes. A study using GAM found that a cutoff value of 0.31 mV in AF was best for predicting <0.5 mV SR–low-voltage substrate.^13^ However, a point-by-point analysis was performed, which could have been affected by undetected map shifts. To address this question, our analysis focused on small and equidistant regions; the mean bipolar voltage within each identically sized area was then computed. Regardless, a similar cutoff value (0.27 mV) was found based on GAMs analysis. Although the results differ slightly from previous studies, the study populations may not be the same. Our study balanced the male-to-female ratio, whereas others did not. The differences observed are likely caused by the role of sex in the outcomes. Moreover, as indicated in Table 3, these cutoffs could be even further adjusted according to the sampled region.

### Sex differences in atrial voltage

Several studies have confirmed that women with AF have lower atrial voltages and a higher incidence of LVAs.^14^^,^^24^^,^^25^ In a magnetic resonance imaging study, the extent of delayed enhancement was greater in women with AF than in men with AF and was thought to represent more severe LA fibrosis.^26^ A recent study showed that older women with paroxysmal AF have more advanced atrial substrates than men and adding low-voltage ablation improves ablation success.^17^ Currently, the exact mechanism underlying sex-related differences in the impact of AF is not fully understood. Compared with men, postmenopausal women with AF have more periarterial adipose tissue, which is associated with a lower atrial voltage.^27^ Studies have shown that female hormones, including progesterone and estrogen, affect the electrical activity of heart muscle cells.^28^ Taken together, these findings partly explain why women—especially among the elderly—exhibit a more advanced atrial substrate than men in early-stage AF, as evidenced by a significantly higher prevalence of LVAs on high-density mapping. 1 study has demonstrated that females exhibit more advanced atrial remodeling as evidenced by high-density voltage mapping, along with a lower mean voltage,^14^ suggesting that hormonal factors play a significant role. The current study demonstrated significant sex-related differences in atrial substrate in patients with AF, characterized by lower voltage and increased complex fractionated signals in females compared with males. Women have been shown to have greater degrees of atrial fibrosis than men in histologic studies including fibrotic markers and on delayed gadolinium enhancement imaging with cardiac magnetic resonance in patients with and without AF.^16^ A small previous electroanatomic study in patients with and without AF did not observe any sex-related differences in atrial electrophysiology.^29^ However, this study used lower-density point-by-point mapping, which may have lacked the resolution and power to detect subtle intergroup differences. Using higher-resolution (smaller electrode spacing) and higher-density mapping, we now show consistent sex-related differences in the AF population at 2 cycle lengths. Notably, these remodeling differences were independent of atrial/ventricular size or sex-specific AF risk-factor prevalence. However, unmeasured factors such as lower atrial wall thickness may potentially account for lower atrial voltages in women,^17^

### Limitations

This study provides novel evidence of a potential causal link among low voltage, rhythm, and sex; however, limitations exist. First, the sample size included in this study was relatively small. Second, the electroanatomic voltage mapping we conducted using the PentaRay catheter; therefore, our findings may not be applicable to other positioning systems and catheters. Furthermore, instead of mapping the low-voltage substrate during native AF and comparing it with SR, we deliberately induced AF and performed voltage mapping after a 10-minute stabilization period. This approach was adopted to prevent any shift in the 3-dimensional anatomic shell. Although the electrophysiological substrate of spontaneous AF may differ from that of induced AF, previous studies have demonstrated a high concordance in the identification of LVAs between the 2 conditions.^30^ Fourth, given that the present cohort consisted predominantly of postmenopausal women, whether these conclusions apply to younger patients requires further research. Finally, because all enrolled subjects were Asian and LA voltage may differ across ethnic groups, additional investigations are required to confirm our findings in other populations.

## Conclusion

The atrial voltage was significantly lower in women than in men, leading to sex-specific differences in bipolar voltage cutoff points. There were significant differences in global and regional voltage distribution when we compared different areas during the same rhythm or different rhythms in the same area. It is possible to establish a new cutoff value for AF with acceptable validity in predicting sinus voltage of <0.5 mV, but gender differences need to be taken into account.

## Disclosures

The authors have no conflicts to disclose.
