# Supplementary material for: Comparative cardiac electrophysiological analysis between sinus rhythm and atrial fibrillation: The correlation of left atrial low-voltage substrate of sex and rhythm
Source: Heart Rhythm O2. 2025 Dec 5;7(2):273–83. doi: 10.1016/j.hroo.2025.11.026 (PMC12925902; doi:10.1016/j.hroo.2025.11.026)
Supplement: Supplementary Material [file mmc1.docx]

**High-density electroanatomic mapping.**

A 6-F decapolar catheter was advanced in the coronary sinus as mapping reference. The patient was cardioverted at the beginning of the study to obtain the first map in SR (avoiding map displacement after the cardioversion). The CARTO system incorporates a TPI (tissue proximity indicator) that monitors impedance matrices to ensure stable endocardial contact of the mapping catheter. To carry out the automatic acquisition of points, a series of filters was included. Multiple bipolar signals (filter setting: 30–500 Hz) were recorded with the PentaRay catheter. During SR, atrial electrograms were captured by setting the window of interest from 50-350 ms, preceding the sharp component of each ventricular QRS complex as a reference (using CS as the reference). High-density bipolar voltage mapping of the LA was performed using a 5-mm filling threshold under conditions of uniform point distribution, with a minimum of 1,500 points acquired per LA map. To avoid poor contact points, we set the interior and exterior projection distance filtering to 5 mm from the geometry surface. To minimize respiratory-related mapping shifts, a respiratory gating method was employed to ensure that anatomical points were acquired only at end-expiration during mechanical ventilation. The Confidence algorithm (Biosense Webster) for tissue proximity indication detection was used to assess whether the tissue-catheter contact at each acquired point was sufficient for electrode-tissue contact. Considering the temporal amplitude variability of the intracardiac electrograms during acquisition, each point was selected by recording the maximal peak-to-peak bipolar voltage amplitude value within the window of interest during 10 consecutive QRS complexes (excluding the QRS interval). LVAs were defined as 0.1- 0.49 mV (peak-to-peak bipolar voltage) and transitional zones as 0.5 to 1.4 mV. Afterward, AF was induced by atrial burst pacing from the distal or mid-coronary sinus at a cycle length of 250- 180ms. Mapping was performed 10 min after AF induction and only if AF was maintained for the entire mapping time.
