# Supplementary material for: Comparative cardiac electrophysiological analysis between sinus rhythm and atrial fibrillation: The correlation of left atrial low-voltage substrate of sex and rhythm
Source: Heart Rhythm O2. 2025 Dec 5;7(2):273–83. doi: 10.1016/j.hroo.2025.11.026 (PMC12925902; doi:10.1016/j.hroo.2025.11.026)

Online Table 1. Baseline characteristics.

| Parameters | Females(n=55) | Males(n=134) | P value |
| --- | --- | --- | --- |
| Age,years | 66.47±5.30 | 60.28±9.03 | 0.110 |
| BMI,KG/m2 | 27.89（24.54，29.39） | 26.85（25.32，29.13） | 0.536 |
| Hypertension,n(%) | 53.3 | 52.2 | 0.935 |
| Diabetes,n(%) | 26.7 | 14.4 | 0.417 |
| Stroke,n(%) | 13.3 | 8.9 | 0.946 |
| Structural heart disease,n(%) | 0 | 5.6 | 0.779 |
| CHD,n(%) | 26.7 | 18.9 | 0.727 |
| AF duration,month | 3（1,12） | 5（1,5） | 0.240 |
| CHA2DS2-VASc score | 3（2,3） | 1（1,3） | 0.006 |
| HAS-BLED score | 2（1,2） | 1（1,2） | 0.150 |
| LAD,mm | 44.54±6.73 | 44.25±4.80 | 0.840 |
| LVEDD,mm | 47.40（42.10,50.90） | 47.0（44.56，51.13） | 0.564 |
| LVEF,% | 62（56,66） | 62（55,65） | 0.826 |
| BNP,pg/ml | 180.31（114.69，335.98） | 134.26（85.50，266.10） | 0.166 |
| CrCL,ml/min/1.73m2 | 89.60（87.29，104.82） | 104.56（88.99，123.68） | 0.034 |

Online Table 2. The number of mean regional LA voltage obtained in each left atrial location.

| Patient | Gender | Anterior | Posterior | Lateral | Septum | Inferior | Roof | Left atrial appendage | Total |
| --- | --- | --- | --- | --- | --- | --- | --- | --- | --- |
| 1 | Male | 8 | 8 | 4 | 9 | 0 | 6 | 9 | 44 |
| 2 | Male | 5 | 7 | 5 | 5 | 3 | 6 | 0 | 31 |
| 3 | Male | 6 | 6 | 2 | 5 | 0 | 6 | 3 | 28 |
| 4 | Male | 9 | 7 | 1 | 4 | 4 | 8 | 6 | 39 |
| 5 | Male | 4 | 9 | 5 | 4 | 6 | 4 | 5 | 37 |
| 6 | Male | 9 | 4 | 7 | 5 | 4 | 9 | 6 | 44 |
| 7 | Male | 7 | 6 | 9 | 8 | 9 | 8 | 3 | 50 |
| 8 | Male | 9 | 7 | 5 | 9 | 7 | 6 | 1 | 44 |
| 9 | Male | 9 | 7 | 7 | 5 | 6 | 6 | 3 | 43 |
| 10 | Male | 9 | 9 | 4 | 4 | 9 | 9 | 1 | 45 |
| 11 | Male | 9 | 9 | 8 | 6 | 8 | 9 | 7 | 56 |
| 12 | Male | 6 | 6 | 2 | 5 | 6 | 7 | 0 | 32 |
| 13 | Male | 9 | 9 | 3 | 5 | 7 | 7 | 3 | 43 |
| 14 | Male | 8 | 3 | 1 | 5 | 4 | 7 | 1 | 29 |
| 15 | Male | 8 | 5 | 2 | 8 | 3 | 8 | 4 | 38 |
| 16 | Female | 8 | 9 | 6 | 9 | 6 | 7 | 6 | 51 |
| 17 | Female | 9 | 6 | 6 | 6 | 7 | 9 | 6 | 49 |
| 18 | Female | 6 | 4 | 4 | 8 | 1 | 8 | 2 | 33 |
| 19 | Female | 9 | 9 | 6 | 7 | 4 | 9 | 4 | 48 |
| 20 | Female | 9 | 9 | 9 | 5 | 0 | 9 | 5 | 46 |
| 21 | Female | 8 | 9 | 3 | 2 | 5 | 5 | 8 | 40 |
| 22 | Female | 7 | 8 | 6 | 5 | 2 | 8 | 8 | 44 |
| 23 | Female | 8 | 7 | 6 | 6 | 1 | 9 | 5 | 42 |
| 24 | Female | 9 | 8 | 5 | 7 | 2 | 6 | 7 | 44 |
| 25 | Female | 9 | 9 | 4 | 7 | 9 | 9 | 5 | 52 |
| 26 | Female | 9 | 9 | 9 | 9 | 7 | 8 | 8 | 59 |
| 27 | Female | 9 | 8 | 9 | 8 | 6 | 8 | 6 | 54 |
| 28 | Female | 6 | 7 | 3 | 1 | 4 | 4 | 2 | 27 |
| 29 | Female | 9 | 8 | 4 | 6 | 3 | 4 | 2 | 36 |
| 30 | Female | 8 | 7 | 2 | 3 | 7 | 7 | 6 | 40 |
| Toal | | 238 | 219 | 147 | 176 | 140 | 216 | 133 | 1268 |

Online Table 3. Predicted voltage in sinus rhythm (mean, minimum and maximum) for each value obtained in atrial fibrillation. As shown, 0.27 mV in atrial fibrillation would correspond with 0.5 mV in sinus rhythm. (CI95% 0.078-1.578 and 0.052-1.198 respectively).

| **A. AF (mV) (mV)** | **Predicted sinus voltage (mean)** | **Predicted sinus voltage (min)** | **Predicted sinus voltage (max)** | 1. **Male**   **AF (mV)** | **Predicted sinus voltage (mean)** | **Predicted sinus voltage (min)** | **Predicted sinus voltage (max)** | **C. Female AF(mV)** | **Predicted sinus voltage (mean)** | **Predicted sinus voltage (min)** | **Predicted sinus voltage (max)** |
| --- | --- | --- | --- | --- | --- | --- | --- | --- | --- | --- | --- |
| 0.050 | 0.248 | 0.211 | 0.292 | 0.050 | 0.453 | 0.373 | 0.550 | 0.050 | 0.202 | 0.163 | 0.250 |
| 0.100 | 0.292 | 0.256 | 0.332 | 0.100 | 0.482 | 0.408 | 0.569 | 0.100 | 0.242 | 0.205 | 0.286 |
| 0.150 | 0.343 | 0.310 | 0.380 | 0.150 | 0.513 | 0.445 | 0.591 | 0.150 | 0.290 | 0.255 | 0.331 |
| 0.200 | 0.402 | 0.369 | 0.437 | 0.200 | 0.545 | 0.482 | 0.616 | 0.200 | 0.346 | 0.309 | 0.387 |
| 0.250 | 0.466 | 0.432 | 0.504 | 0.250 | 0.579 | 0.520 | 0.645 | 0.250 | 0.409 | 0.367 | 0.457 |
| 0.300 | 0.535 | 0.495 | 0.579 | 0.300 | 0.614 | 0.557 | 0.678 | 0.300 | 0.478 | 0.426 | 0.536 |
| 0.350 | 0.604 | 0.556 | 0.656 | 0.350 | 0.650 | 0.592 | 0.714 | 0.350 | 0.549 | 0.487 | 0.617 |
| 0.400 | 0.669 | 0.615 | 0.728 | 0.400 | 0.686 | 0.626 | 0.752 | 0.400 | 0.617 | 0.548 | 0.696 |
| 0.450 | 0.727 | 0.668 | 0.790 | 0.450 | 0.722 | 0.659 | 0.790 | 0.450 | 0.680 | 0.602 | 0.768 |
| 0.500 | 0.774 | 0.712 | 0.842 | 0.500 | 0.756 | 0.689 | 0.829 | 0.500 | 0.732 | 0.645 | 0.831 |
| 0.550 | 0.811 | 0.744 | 0.884 | 0.550 | 0.790 | 0.718 | 0.868 | 0.550 | 0.773 | 0.675 | 0.884 |
| 0.600 | 0.838 | 0.765 | 0.917 | 0.600 | 0.822 | 0.746 | 0.906 | 0.600 | 0.801 | 0.693 | 0.925 |
| 0.650 | 0.855 | 0.776 | 0.943 | 0.650 | 0.853 | 0.772 | 0.944 | 0.650 | 0.816 | 0.700 | 0.951 |
| 0.700 | 0.867 | 0.782 | 0.961 | 0.700 | 0.883 | 0.797 | 0.980 | 0.700 | 0.822 | 0.699 | 0.967 |
| 0.750 | 0.875 | 0.785 | 0.975 | 0.750 | 0.913 | 0.821 | 1.016 | 0.750 | 0.821 | 0.692 | 0.975 |
| 0.800 | 0.882 | 0.788 | 0.987 | 0.800 | 0.942 | 0.844 | 1.052 | 0.800 | 0.816 | 0.679 | 0.980 |
| 0.850 | 0.890 | 0.792 | 1.000 | 0.850 | 0.972 | 0.867 | 1.089 | 0.850 | 0.809 | 0.665 | 0.984 |
| 0.900 | 0.901 | 0.798 | 1.016 | 0.900 | 1.001 | 0.891 | 1.126 | 0.900 | 0.802 | 0.650 | 0.989 |
| 0.950 | 0.914 | 0.806 | 1.037 | 0.950 | 1.032 | 0.914 | 1.164 | 0.950 | 0.797 | 0.637 | 0.997 |
| 1.000 | 0.932 | 0.816 | 1.064 | 1.000 | 1.062 | 0.938 | 1.203 | 1.000 | 0.795 | 0.628 | 1.006 |
| 1.050 | 0.953 | 0.830 | 1.096 | 1.050 | 1.094 | 0.963 | 1.242 | 1.050 | 0.797 | 0.623 | 1.019 |
| 1.100 | 0.979 | 0.846 | 1.132 | 1.100 | 1.125 | 0.987 | 1.283 | 1.100 | 0.803 | 0.623 | 1.036 |
| 1.150 | 1.008 | 0.867 | 1.172 | 1.150 | 1.158 | 1.012 | 1.325 | 1.150 | 0.814 | 0.627 | 1.057 |
| 1.200 | 1.040 | 0.891 | 1.215 | 1.200 | 1.190 | 1.036 | 1.368 | 1.200 | 0.830 | 0.635 | 1.085 |
| 1.250 | 1.075 | 0.918 | 1.259 | 1.250 | 1.224 | 1.061 | 1.411 | 1.250 | 0.851 | 0.646 | 1.120 |
| 1.300 | 1.112 | 0.948 | 1.305 | 1.300 | 1.257 | 1.085 | 1.455 | 1.300 | 0.877 | 0.661 | 1.163 |
| 1.350 | 1.151 | 0.979 | 1.354 | 1.350 | 1.290 | 1.110 | 1.500 | 1.350 | 0.908 | 0.679 | 1.215 |
| 1.400 | 1.191 | 1.010 | 1.404 | 1.400 | 1.324 | 1.134 | 1.544 | 1.400 | 0.944 | 0.699 | 1.274 |
| 1.450 | 1.232 | 1.041 | 1.458 | 1.450 | 1.357 | 1.158 | 1.590 | 1.450 | 0.983 | 0.721 | 1.341 |
| 1.500 | 1.273 | 1.069 | 1.514 | 1.500 | 1.391 | 1.182 | 1.636 | 1.500 | 1.026 | 0.745 | 1.414 |
| 1.550 | 1.314 | 1.096 | 1.574 | 1.550 | 1.424 | 1.204 | 1.685 | 1.550 | 1.072 | 0.771 | 1.492 |
| 1.600 | 1.355 | 1.122 | 1.635 | 1.600 | 1.458 | 1.226 | 1.734 | 1.600 | 1.120 | 0.797 | 1.574 |
| 1.650 | 1.395 | 1.147 | 1.698 | 1.650 | 1.492 | 1.246 | 1.785 | 1.650 | 1.169 | 0.824 | 1.659 |
| 1.700 | 1.436 | 1.172 | 1.760 | 1.700 | 1.526 | 1.267 | 1.838 | 1.700 | 1.217 | 0.848 | 1.747 |
| 1.750 | 1.477 | 1.196 | 1.823 | 1.750 | 1.560 | 1.287 | 1.891 | 1.750 | 1.264 | 0.870 | 1.835 |
| 1.800 | 1.517 | 1.221 | 1.885 | 1.800 | 1.594 | 1.307 | 1.944 | 1.800 | 1.308 | 0.889 | 1.924 |
| 1.850 | 1.557 | 1.244 | 1.948 | 1.850 | 1.628 | 1.326 | 1.998 | 1.850 | 1.348 | 0.902 | 2.012 |
| 1.900 | 1.596 | 1.266 | 2.012 | 1.900 | 1.661 | 1.344 | 2.053 | 1.900 | 1.383 | 0.911 | 2.100 |
| 1.950 | 1.634 | 1.285 | 2.078 | 1.950 | 1.694 | 1.360 | 2.109 | 1.950 | 1.413 | 0.914 | 2.184 |
| 2.000 | 1.671 | 1.302 | 2.146 | 2.000 | 1.725 | 1.375 | 2.165 | 2.000 | 1.437 | 0.912 | 2.264 |
| 2.050 | 1.707 | 1.315 | 2.215 | 2.050 | 1.755 | 1.388 | 2.220 | 2.050 | 1.454 | 0.905 | 2.337 |
| 2.100 | 1.741 | 1.326 | 2.285 | 2.100 | 1.784 | 1.400 | 2.273 | 2.100 | 1.465 | 0.894 | 2.400 |
| 2.150 | 1.772 | 1.335 | 2.352 | 2.150 | 1.810 | 1.410 | 2.324 | 2.150 | 1.469 | 0.880 | 2.452 |
| 2.200 | 1.801 | 1.343 | 2.415 | 2.200 | 1.834 | 1.419 | 2.371 | 2.200 | 1.466 | 0.863 | 2.491 |
| 2.250 | 1.826 | 1.349 | 2.472 | 2.250 | 1.855 | 1.426 | 2.414 | 2.250 | 1.456 | 0.843 | 2.515 |
| 2.300 | 1.847 | 1.353 | 2.521 | 2.300 | 1.874 | 1.431 | 2.453 | 2.300 | 1.441 | 0.822 | 2.527 |
| 2.350 | 1.864 | 1.356 | 2.563 | 2.350 | 1.889 | 1.433 | 2.489 | 2.350 | 1.421 | 0.799 | 2.527 |
| 2.400 | 1.876 | 1.356 | 2.594 | 2.400 | 1.900 | 1.433 | 2.519 | 2.400 | 1.396 | 0.774 | 2.518 |
| 2.450 | 1.883 | 1.354 | 2.617 | 2.450 | 1.909 | 1.431 | 2.546 | 2.450 | 1.367 | 0.746 | 2.503 |
| 2.500 | 1.884 | 1.349 | 2.631 | 2.500 | 1.913 | 1.425 | 2.568 | 2.500 | 1.335 | 0.717 | 2.485 |
| 2.550 | 1.880 | 1.340 | 2.637 | 2.550 | 1.914 | 1.416 | 2.586 | 2.550 | 1.301 | 0.687 | 2.465 |
| 2.600 | 1.869 | 1.326 | 2.635 | 2.600 | 1.911 | 1.404 | 2.599 | 2.600 | 1.266 | 0.655 | 2.446 |
| 2.650 | 1.854 | 1.308 | 2.627 | 2.650 | 1.904 | 1.390 | 2.608 | 2.650 | 1.229 | 0.623 | 2.427 |
| 2.700 | 1.832 | 1.284 | 2.614 | 2.700 | 1.893 | 1.373 | 2.611 | 2.700 | 1.193 | 0.590 | 2.410 |
| 2.750 | 1.805 | 1.256 | 2.595 | 2.750 | 1.879 | 1.354 | 2.609 | 2.750 | 1.156 | 0.559 | 2.393 |
| 2.800 | 1.773 | 1.223 | 2.571 | 2.800 | 1.862 | 1.333 | 2.602 | 2.800 | 1.121 | 0.529 | 2.376 |
| 2.850 | 1.737 | 1.187 | 2.543 | 2.850 | 1.842 | 1.310 | 2.591 | 2.850 | 1.087 | 0.501 | 2.357 |
| 2.900 | 1.697 | 1.147 | 2.509 | 2.900 | 1.819 | 1.285 | 2.576 | 2.900 | 1.054 | 0.476 | 2.335 |
| 2.950 | 1.653 | 1.107 | 2.470 | 2.950 | 1.794 | 1.258 | 2.557 | 2.950 | 1.023 | 0.453 | 2.308 |
| 3.000 | 1.607 | 1.065 | 2.426 | 3.000 | 1.766 | 1.230 | 2.535 | 3.000 | 0.994 | 0.434 | 2.277 |
| 3.050 | 1.559 | 1.023 | 2.377 | 3.050 | 1.736 | 1.199 | 2.512 | 3.050 | 0.967 | 0.417 | 2.241 |
| 3.100 | 1.510 | 0.982 | 2.323 | 3.100 | 1.704 | 1.167 | 2.488 | 3.100 | 0.942 | 0.403 | 2.201 |
| 3.150 | 1.461 | 0.941 | 2.266 | 3.150 | 1.671 | 1.133 | 2.463 | 3.150 | 0.919 | 0.391 | 2.157 |
| 3.200 | 1.411 | 0.903 | 2.205 | 3.200 | 1.636 | 1.098 | 2.439 | 3.200 | 0.898 | 0.382 | 2.110 |
| 3.250 | 1.362 | 0.866 | 2.142 | 3.250 | 1.601 | 1.061 | 2.414 | 3.250 | 0.878 | 0.374 | 2.061 |
| 3.300 | 1.314 | 0.831 | 2.078 | 3.300 | 1.564 | 1.024 | 2.390 | 3.300 | 0.861 | 0.368 | 2.013 |
| 3.350 | 1.268 | 0.798 | 2.014 | 3.350 | 1.527 | 0.986 | 2.366 | 3.350 | 0.845 | 0.364 | 1.965 |
| 3.400 | 1.223 | 0.766 | 1.951 | 3.400 | 1.490 | 0.948 | 2.343 | 3.400 | 0.832 | 0.360 | 1.919 |
| 3.450 | 1.180 | 0.737 | 1.891 | 3.450 | 1.452 | 0.909 | 2.319 | 3.450 | 0.819 | 0.358 | 1.876 |
| 3.500 | 1.140 | 0.709 | 1.833 | 3.500 | 1.414 | 0.871 | 2.297 | 3.500 | 0.809 | 0.356 | 1.836 |
| 3.550 | 1.102 | 0.682 | 1.781 | 3.550 | 1.377 | 0.833 | 2.275 | 3.550 | 0.800 | 0.355 | 1.801 |
| 3.600 | 1.067 | 0.656 | 1.733 | 3.600 | 1.339 | 0.795 | 2.255 | 3.600 | 0.792 | 0.355 | 1.770 |
| 3.650 | 1.034 | 0.632 | 1.691 | 3.650 | 1.301 | 0.757 | 2.236 | 3.650 | 0.787 | 0.355 | 1.745 |
| 3.700 | 1.004 | 0.609 | 1.655 | 3.700 | 1.264 | 0.720 | 2.220 | 3.700 | 0.783 | 0.355 | 1.726 |
| 3.750 | 0.976 | 0.587 | 1.624 | 3.750 | 1.228 | 0.683 | 2.206 | 3.750 | 0.780 | 0.355 | 1.712 |
| 3.800 | 0.951 | 0.566 | 1.600 | 3.800 | 1.191 | 0.647 | 2.195 | 3.800 | 0.779 | 0.356 | 1.703 |
| 3.850 | 0.929 | 0.546 | 1.580 | 3.850 | 1.156 | 0.611 | 2.187 | 3.850 | 0.780 | 0.358 | 1.701 |
| 3.900 | 0.909 | 0.528 | 1.566 | 3.900 | 1.121 | 0.575 | 2.184 | 3.900 | 0.782 | 0.359 | 1.704 |
| 3.950 | 0.892 | 0.512 | 1.556 | 3.950 | 1.086 | 0.540 | 2.184 | 3.950 | 0.786 | 0.361 | 1.712 |
| 4.000 | 0.878 | 0.497 | 1.550 | 4.000 | 1.053 | 0.506 | 2.189 | 4.000 | 0.791 | 0.363 | 1.726 |
| 4.050 | 0.866 | 0.485 | 1.547 | 4.050 | 1.020 | 0.473 | 2.198 | 4.050 | 0.798 | 0.365 | 1.744 |
| 4.100 | 0.857 | 0.474 | 1.548 | 4.100 | 0.988 | 0.441 | 2.211 | 4.100 | 0.806 | 0.368 | 1.766 |
| 4.150 | 0.850 | 0.466 | 1.552 | 4.150 | 0.957 | 0.410 | 2.229 | 4.150 | 0.816 | 0.372 | 1.792 |
| 4.200 | 0.845 | 0.459 | 1.558 | 4.200 | 0.926 | 0.381 | 2.251 | 4.200 | 0.827 | 0.376 | 1.821 |
| 4.250 | 0.843 | 0.453 | 1.567 | 4.250 | 0.897 | 0.353 | 2.278 | 4.250 | 0.840 | 0.381 | 1.853 |
| 4.300 | 0.843 | 0.449 | 1.580 | 4.300 | 0.868 | 0.326 | 2.308 | 4.300 | 0.853 | 0.386 | 1.887 |
| 4.350 | 0.844 | 0.447 | 1.596 | 4.350 | 0.840 | 0.301 | 2.342 | 4.350 | 0.868 | 0.392 | 1.925 |
| 4.400 | 0.848 | 0.445 | 1.616 | 4.400 | 0.813 | 0.278 | 2.380 | 4.400 | 0.884 | 0.397 | 1.966 |
| 4.450 | 0.853 | 0.444 | 1.641 | 4.450 | 0.787 | 0.256 | 2.421 | 4.450 | 0.901 | 0.404 | 2.011 |
| 4.500 | 0.860 | 0.443 | 1.671 | 4.500 | 0.761 | 0.235 | 2.465 | 4.500 | 0.918 | 0.409 | 2.060 |
| 4.550 | 0.869 | 0.442 | 1.706 | 4.550 | 0.737 | 0.216 | 2.512 | 4.550 | 0.937 | 0.415 | 2.115 |
| 4.600 | 0.878 | 0.441 | 1.749 | 4.600 | 0.713 | 0.199 | 2.561 | 4.600 | 0.956 | 0.420 | 2.175 |
| 4.650 | 0.890 | 0.440 | 1.799 | 4.650 | 0.690 | 0.182 | 2.613 | 4.650 | 0.975 | 0.424 | 2.242 |
| 4.700 | 0.902 | 0.438 | 1.857 | 4.700 | 0.668 | 0.167 | 2.668 | 4.700 | 0.995 | 0.427 | 2.316 |
| 4.750 | 0.915 | 0.435 | 1.923 | 4.750 | 0.646 | 0.153 | 2.725 | 4.750 | 1.015 | 0.429 | 2.398 |
| 4.800 | 0.929 | 0.432 | 1.999 | 4.800 | 0.626 | 0.141 | 2.785 | 4.800 | 1.035 | 0.430 | 2.490 |
| 4.850 | 0.944 | 0.428 | 2.084 | 4.850 | 0.605 | 0.129 | 2.847 | 4.850 | 1.055 | 0.429 | 2.591 |
| 4.900 | 0.959 | 0.423 | 2.178 | 4.900 | 0.586 | 0.118 | 2.912 | 4.900 | 1.074 | 0.427 | 2.703 |
| 4.950 | 0.975 | 0.417 | 2.281 | 4.950 | 0.567 | 0.108 | 2.979 | 4.950 | 1.094 | 0.423 | 2.825 |
| 5.000 | 0.991 | 0.410 | 2.393 | 5.000 | 0.549 | 0.099 | 3.048 | 5.000 | 1.112 | 0.418 | 2.959 |

**Online Figure 1 .The thresholds corresponding to various parts of the left atrium.**

Anterior Posterior Lateral


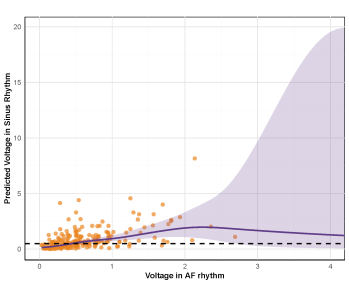

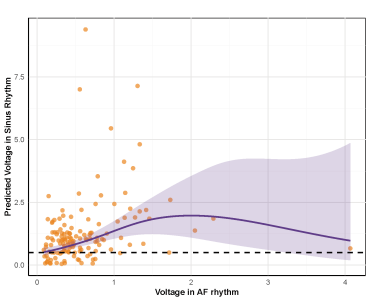

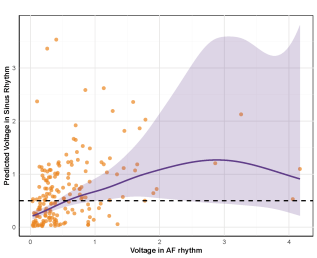


Septum Inferior Roof


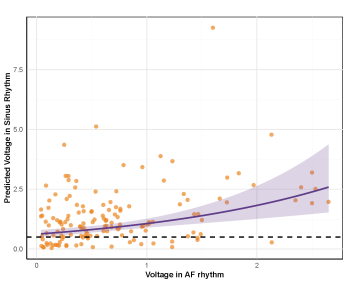

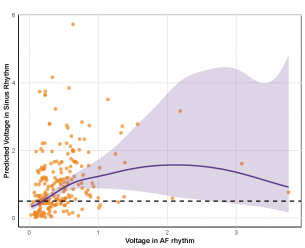

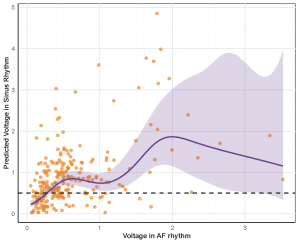


Left atrial appendage


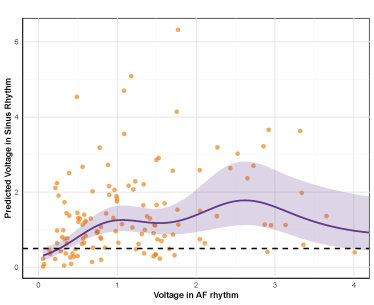

Supplement: Supplementary Table [file mmc2.docx]
